# Determinants of Anishinabeck infant and early childhood growth trajectories in Northwestern Ontario, Canada: a cohort study

**DOI:** 10.1186/s12887-023-04449-5

**Published:** 2023-12-19

**Authors:** Hiliary Monteith, Mary Mamakeesick, Joan Rae, Tracey Galloway, Stewart B. Harris, Catherine Birken, Jill Hamilton, Jonathon L. Maguire, Patricia Parkin, Bernard Zinman, Anthony J. G. Hanley

**Affiliations:** 1 Department of Nutritional Sciences, Temerty Faculty of Medicine, University of Toronto, University of Toronto Medical Sciences Building, 5Th Floor, Room 5253A, 1 King’s College Circle, Toronto, ON M5S 1A8 Canada; 2 Sandy Lake First Nation, Sandy Lake, ON P0V 1V0 Canada; 3 Department of Anthropology, University of Toronto Mississauga Campus, Terrence Donnelly Health Sciences Complex, Room 354, 3359 Mississauga Rd, Mississauga, ON L5L 1C6 Canada; 4 Schulich School of Medicine and Dentistry, Western Centre for Public Health & Family Medicine, Western University, 1465 Richmond St, London, N6G 2M1 ON Canada; 5 Department of Pediatrics, Temerty Faculty of Medicine, University of Toronto, University of Toronto Medical Sciences Building, 5Th Floor, Room 5271, 1 King’s College Circle, Toronto, ON M5S 1A8 Canada; 6 Division of Endocrinology, Hospital for Sick Children, Department of Nutritional Sciences, University of Toronto, 555 University Ave, Toronto, ON M5S 1X8 Canada; 7 Lunenfeld Tanenbaum Research Institute, Mount Sinai Hospital, University of Toronto, 600 University Ave, Toronto, ON M5G 1X5 Canada

**Keywords:** Indigenous health, Child growth, Infant nutrition, Diabetes, Obesity, First Nations, Maternal health, Breastfeeding

## Abstract

**Background:**

The Developmental Origins of Health and Disease (DOHaD) paradigm emphasizes the significance of early life factors for the prevention of chronic health conditions, like type 2 diabetes (T2DM) and obesity, which disproportionately affect First Nations communities in Canada. Despite increasing DOHaD research related to maternal health during pregnancy, early childhood growth patterns, and infant feeding practices with many populations, data from First Nations communities in Canada are limited. In partnership with Sandy Lake First Nation, the aims of this project were to characterize birthweights and growth patterns of First Nations infants/children over the first 6 years of life and to study the impact of maternal and infant social and behavioral factors on birthweight and growth trajectories.

**Methods:**

We recruited 194 families through community announcements and clinic visits. Infant/child length/height and weight were measured at 1 and 2 weeks; 1, 2, 6, 12, and 18 months; and 2, 3, 4, 5 and 6 years. Maternal and infant/child questionnaires captured data about health, nutrition, and social support. Weight-for-Age z-score (WAZ), Height-for-Age z-score (HAZ), and BMI-for-Age z-score (BAZ) were calculated using WHO reference standards and trajectories were analyzed using generalized additive models. Generalized estimating equations and logistic regression were used to determine associations between exposures and outcomes.

**Results:**

WAZ and BAZ were above the WHO mean and increased with age until age 6 years. Generalized estimating equations indicated that WAZ was positively associated with age (0.152; 95% CI 0.014, 0.29), HAZ was positively associated with birthweight (0.155; 95% CI 0.035, 0.275), and BAZ was positively associated with caregiver’s BMI (0.049; 95% CI 0.004, 0.090). There was an increased odds of rapid weight gain (RWG) with exposure to gestational diabetes (OR: 7.47, 95% CI 1.68, 46.22). Almost 70% of parents initiated breastfeeding, and breastfeeding initiation was modestly associated with lower WAZ (-0.18; 95% CI -0.64, 0.28) and BAZ (-0.23; 95% CI -0.79, 0.34).

**Conclusions:**

This work highlights early life factors that may contribute to T2DM etiology and can be used to support community and Indigenous-led prevention strategies.

## Background

The Developmental Origins of Health and Disease (DOHaD) concept was initially proposed by Barker and Bagby [1], who identified connections between low birthweight and increased chronic disease risk many years later; however, the idea that chronic disease is related to experiences in infancy was introduced much earlier [2, 3]. DOHaD has emerged as an important area of study given the potential for mitigating disease risk through interventions implemented before birth and in the earliest years of life [4]. There is now substantial evidence indicating that fetal and infant development and associated phenotypic phenomena are connected to health outcomes later in life [1, 5].

Indigenous communities in Canada, which include First Nations, Inuit, and Métis Peoples with several unique cultures therein, have long understood health as strongly connected to kinship and relationships. Intergenerational health refers to health shared across a generation (eg. parent and child) and is an important cultural perspective for many Indigenous communities with consideration of health impacts on future generations. The Haudenosaunee Seventh Generation Principle refers to the need to consider the impact on the next seven generations in all decision-making [6]. In addition, the National Collaborating Centre for Indigenous Health describes the integrated life course as an important framework when considering the social determinants of health where all life stages and age groups are included [7]. In light of this context, DOHaD research aligns well with many Indigenous health knowledge frameworks and therefore may contribute meaningful information for Indigenous communities and future generations.

Negative health outcomes and mortality are often increased for First Nations compared to non-First Nations in Canada [8–10], which is strongly connected, directly and indirectly, to colonial legacies and racism in Canada, often enacted through a lack of cultural safety and access to medical care within the healthcare system [11]. Prenatal exposures, birthweight, and other factors occurring in early life, including maternal nutrition and gestational diabetes (GDM), may influence long-term risk of T2DM and obesity, in part through detrimental effects on infant growth [5]. Previous studies have demonstrated a relationship between GDM and T2DM exposure in utero and risk of diabetes and overweight/obesity for the child later in life [12, 13]. In addition, it is known that birthweight is associated with risk for T2DM [5, 14]. Dyck et al. [14] demonstrated that for First Nations women who themselves had high-birthweight, the risk for GDM and T2DM is significantly elevated. Infant and childhood growth trajectories may also have a significant relationship with the incidence of early onset of T2DM and overweight and obesity in adolescence and adulthood. Geserick et al. [15] conducted a prospective analysis that demonstrated that 90% of children who had obesity at three years of age, had overweight or obesity in adolescence and the greatest acceleration of BMI occurred between two and six years of age, indicating that rapid weight gain (RWG) among preschoolers (aged 2–6) may contribute to future overweight and obesity. Previous studies have considered infant growth trajectories and associations with birthweight, T2DM, overweight/obesity, blood pressure, and infant feeding [16–20]; however, few have considered Indigenous growth trajectories, with only one known published study in Canada [21]. Given documented high birthweights and higher prevalence rates of T2DM, overweight/obesity, cardiovascular disease, maternal smoking, GDM, and low breastfeeding rates for Indigenous populations in Canada [22–24], it is imperative that growth trajectories of Indigenous peoples be characterized and analyzed to explore possible associations that are unique to Indigenous communities. This information will inform culturally appropriate and relevant health promotion policies and interventions to support optimal growth. Further, this information will fill a significant gap in the literature and aligns with community perspectives of intergenerational health and wellbeing where health and wellbeing are shared across life stages.

On this background, the *objectives* of the current study were 1) to characterize birthweights and growth patterns of Indigenous infants and children during the first six years of life, and 2) to study the impact of maternal and infant factors (e.g. GDM and breastfeeding) on growth patterns during this period. We hypothesized that growth patterns would be characterized by mean z-scores above the WHO mean, with a faster growth rate, and that RWG and early childhood overweight/obesity will be associated with caregiver BMI, GDM and infant feeding practices (breastfeeding compared to not).

### Study design and participants

Neh gaaw saga’igan (Sandy Lake) is a remote Anishinabeck (Oji-Cree) community located in Northwestern Ontario, with a population of approximately 2000 people [25]. Building on a long-standing relationship between the University of Toronto and Sandy Lake First Nation, and previous research [26, 27], we collaborated with the Sandy Lake Health and Diabetes Project (SLHDP) to identify the impact of social, environmental, and behavioral risk factors on infant growth trajectories with a specific focus on maternal and infant/child nutrition and diabetes risk. This project followed the principles of OCAP® with guidance from Sandy Lake First Nation leadership, emphasizing a need to explore relationships between childhood and diabetes risk to protect future generations [27].

Families with infants between birth and six years of age were eligible and invited to participate in this study from July 1, 2013 to June 30, 2019. Families not planning to reside in Sandy Lake for the duration of the project and pregnant women not permanently residing in the community were excluded, as follow-up visits required families to be in the community for the duration of the study. Additional exclusion criteria were not incorporated at enrollment to ensure that the cohort would be as representative as possible; however, there were 17 children born prior to 37 weeks gestation who were excluded from analysis, since premature infants may have growth differences that would impact the overall population growth trajectory [28]. In response to recruitment challenges, we enrolled children under 6 years of age; however, most initial assessments were completed when the child was under 2 years old. At recruitment interviews, the study was described in detail, questions answered, and written informed consent obtained. Ethical approval was provided through the University of Toronto Research Ethics Board and the Sandy Lake First Nation Chief and Council. To enhance recruitment, this study was promoted through radio announcements and advertisements in high traffic areas of the community (ie. Nursing Station, band office, stores).

All pregnant women in Sandy Lake travel by air to Sioux Lookout, Thunder Bay, or Winnipeg at 36–37 weeks of gestation for hospital delivery, which is standard clinical practice in this region. Healthy mothers and babies are discharged and return to Sandy Lake by plane two to three days postpartum; however, longer stays in hospital are required with pregnancy and/or neonatal complications. Once in the community, standard neonatal and post-natal care are provided by the Public Health Nurse’s office and Nishtum (Early Childhood Centre) and includes well baby checks and immunizations at the following ages: one and two weeks; one, two, four, six, nine, 12, 15 and 18 months; and two, three, four, five, and six years (Rourke guidelines) [29]. Initially, we planned to coordinate assessments with the Public Health Nurse’s office to maximize convenience for participants; however, participants felt that the frequency and number of assessments was cumbersome. For this reason, research assessments were adjusted to one and two weeks; one, two, six, 12, 18 months; and two, three, four, five, and six years. The study team identified improved adherence to the follow-up schedule and satisfaction from participants with this modification.

## Data collection

### Anthropometric measurements

The Sandy Lake research team received training on infant and toddler anthropometric measurement techniques with pediatric co-investigators at the Hospital for Sick Children in Toronto, Canada. Infant/child and caregiver weight was measured using a calibrated electronic scale appropriate for age (infants were measured using a digital pediatric tray scale; older children and adults with a mechanical floor scale). Children older than two years and adults wore light indoor clothing and removed shoes. Recumbent length was measured for children under two years old using a length board. Standing heights were collected for children older than two years and adults using a stadiometer. Measurements were completed twice, and the average used in the analysis. For all equipment, a regular schedule of equipment maintenance and calibration was followed.

### Questionnaires

Maternal and infant/child socio-ecological, behavioral, and metabolic determinants were collected using standardized, interviewer-administered questionnaires. The questionnaires were modified from the TARGet Kids! Nutritional Health Questionnaire [30], a detailed questionnaire adapted from the Canadian Community Health Survey (CCHS) and Canadian Health Measures Survey (CHMS); questions previously used with the SLHDP were also included [27].

Questions specific to infant and child nutrition included exclusivity and duration of breastfeeding, bottle use, introduction and volume of cow’s milk / formula, solid foods, and consumption of sweet drinks. Childhood health conditions were also recorded. Parent-reported health factors included biological parent history of chronic disease (including family history of diabetes), pre-pregnancy weight and T2DM, pregnancy complications such as GDM and preeclampsia, alcohol and drug use during pregnancy, and cigarette smoking during pregnancy or in the home. Questions specific to socioeconomic status included information about family and social support, education, and food insecurity. In Spring 2012, the proposed instruments were pilot tested in Sandy Lake to ensure cultural relevance, suitability, and clarity for the Sandy Lake community. Questionnaires were modified based on feedback from key community informants and pilot study participants. Questionnaires were administered at initial enrollment and at each follow-up visit.

### Statistical analysis

All statistical analyses were conducted using R Software [31]. HAZ, WAZ, and BAZ were calculated using WHO reference data [32, 33]. Z-scores were included as continuous variables in statistical analysis and growth trajectories were plotted using WHO reference data [34–36]. Descriptive statistics were conducted to provide a summary of the sample.

Generalized additive models (GAMs), an extension of generalized linear models that can accommodate non-linearity using smooth functions [37], were used to characterize growth trajectories. The model equation is Y_i_ = β_o_ + ∑s_j_(x_ji_) + є_i,,_ where Y represents the outcome (eg. WAZ) of the ith individual, x equals the predictor variable for the ith child at time j, ∑s_j_ is the sum of the smooth functions, and є is the error term. Splines use basis functions to account for the amount of smoothness in the data and can be adapted for model fit [37]. We used nine basis functions in our analysis as this provided balanced smoothness to help avoid over and underfitting the model.

Cross-sectional analyses using logistic regression models evaluated exposures associated with high birthweight, GDM, and RWG as dichotomous outcome variables. High birthweight was defined as > 4000 g [38]. RWG is generally defined as > 0.67 SD increase in z-score between two measurements in the first two years of life [39]; however, other studies have defined extreme RWG as > 1.28 SD increase [40]. These changes in z-scores correspond to crossing one and two centile bands respectively. As preliminary analysis identified substantial RWG in our sample, we defined a third category of RWG as an increase > 2 SD ("extreme RWG") as a sensitivity analysis. Exposures of interest included maternal factors (e.g. GDM, social factors) and infant health and nutrition (e.g. birthweight, breastfeeding initiation). Potential confounders were identified based on prior literature, biological considerations, and possible influence on sample characteristics.

Longitudinal associations (e.g. associations over time) were assessed using generalized estimating equations (GEE). Outcomes of interest included weight-for-age, height-for-age, and BMI-for-age z-scores. Exposures of interest included breastfeeding initiation, bottle feeding initiation, birthweight, child’s age, caregiver BMI at each visit, diabetes during pregnancy, RWG, maternal weight change (pre and post pregnancy), and alcohol, cigarettes, and other drugs during pregnancy. Models were adjusted for confounders determined a priori based on the literature.

GEE models are similar to regular regression models, although they allow for the use of repeated measures within the same individual. GEE models also allow varying numbers of observations per person and varying timing of those observations among individuals; therefore, missed observations and mis-timed visits are not as problematic as in other types of analyses [41]. This is particularly important to consider for this modified birth cohort as there were missing data from incomplete or missed visits with health assessments in the community for various socio-ecological reasons.

Our sample size of 150 infants and children allowed us to detect odds ratios (ORs) in logistic regression models of 1.5 and 1.7 with a statistical power of 57 and 80% (*p* = 0.05), respectively, per SD increase in continuously distributed exposures.

## Results

This cohort included 194 children; however, after data cleaning for pre-term births and child’s age, we included 150 children in the analyses with initial and repeated measurements from birth to six years old. Most children were brought into research visits with their parent; however, other caregivers were also involved. Most mothers reported being between 20 and 35 years old (77.2%) (Table 1). There were slightly more girls (53.3%) involved in this study compared to boys (46.7%).

**Table 1:** Baseline descriptive infant/child and maternal characteristics

**Infant/Child Characteristics**		**Prevalence (n (%))**
Sex (*n* = 150)	Female	80 (53.3%)
	Male	70 (46.7%)
Birthweight (*n* = 144)	< 2500 g	2 (1.4%)
	≥ 2500 ≤ 4000 g	117 (81.2%)
	> 4000 g	25 (17.4%)
Breastfeeding Initiation (*n* = 150)	Yes	103 (68.7%)
	No	47 (31.3%)
Breastfeeding Duration (*n* = 31)	< 2 months	5 (16.1%)
	> 2 months and < 6 months	10 (32.3%)
	> 6 months	16 (51.6%)
Bottle feeding Initiation^**a**^(*n* = 67)	Yes	56 (83.6%)
	No	11 (16.4%)
Maternal Age (*n* = 136)	< 20 years	19 (14.0%)
	20-35 years	105 (77.2%)
	> 35 years	12 (8.8%)
Paternal Age (*n* = 115)	< 20 years	10 (8.7%)
	20-35 years	84 (73.0%)
	> 35 years	21 (18.3%)
Maternal Employment (*n* = 125)	Employed or on social assistance	17 (13.6)
	Unemployed	108 (86.4%)
Paternal Employment (*n* = 112)	Employed or on social assistance	44 (39.3%)
	Unemployed	68 (60.7%)
Cigarette Smoking at home (*n* = 68)	Yes	58 (85.3%)
	No	10 (14.7%)
Child’s Birthplace (*n* = 149)	Sandy Lake	3 (2%)
	Sioux Lookout	69 (46.3%)
	Thunder Bay	7 (4.7%)
	Winnipeg	67 (45%)
	Other	3 (2%)
**Maternal Exposure**		**Prevalence (n (%))**
Anemia during pregnancy (*n* = 144)	Yes	36 (25.0%)
	No	97 (67.4%)
	Unknown	11 (7.6%)
High Blood Pressure during pregnancy (*n* = 146)	Yes	15 (10.3%)
	No	123 (84.2%)
	Unknown	8 (5.5%)
Gestational Diabetes (*n* = 147)	Yes	31 (21.1%)
	No	116 (78.9%)
Supplements during pregnancy (*n* = 150)	Yes	135 (90.0%)
	No	15 (10.0%)
Unprescribed Meds during pregnancy (*n* = 144)	Yes	42 (29.2%)
	No	102 (70.8%)
Cigarette Smoking during pregnancy (*n* = 144)	Yes	95 (66.0%)
	No	49 (34.0%)
Alcohol during pregnancy (*n* = 142)	Yes	11 (7.7%)
	No	131 (92.3%)
Caregiver BMI (*n* = 70)	Overweight/Obese	52 (74.3%)
	Target	18 (25.7%)

The total n in this study after data cleaning was 150. Therefore, *n* = 150 signifies 100% participation in the questionnaire

^a^Bottle feeding initiation includes any bottle feeding, irrespective of breastfeeding status

Approximately 98% of deliveries occurred in hospitals outside of Sandy Lake and almost 70% of mothers reported initiating breastfeeding. Within this group, 51% were born in large population centers (Winnipeg and Thunder Bay), 45% in a small population center (Sioux Lookout), and 4% in rural areas, including Sandy Lake and surrounding communities. Over 20% of mothers reported having GDM and over 10% and 25% of mothers had hypertension and anemia during pregnancy, respectively.

**Fig. 1:**
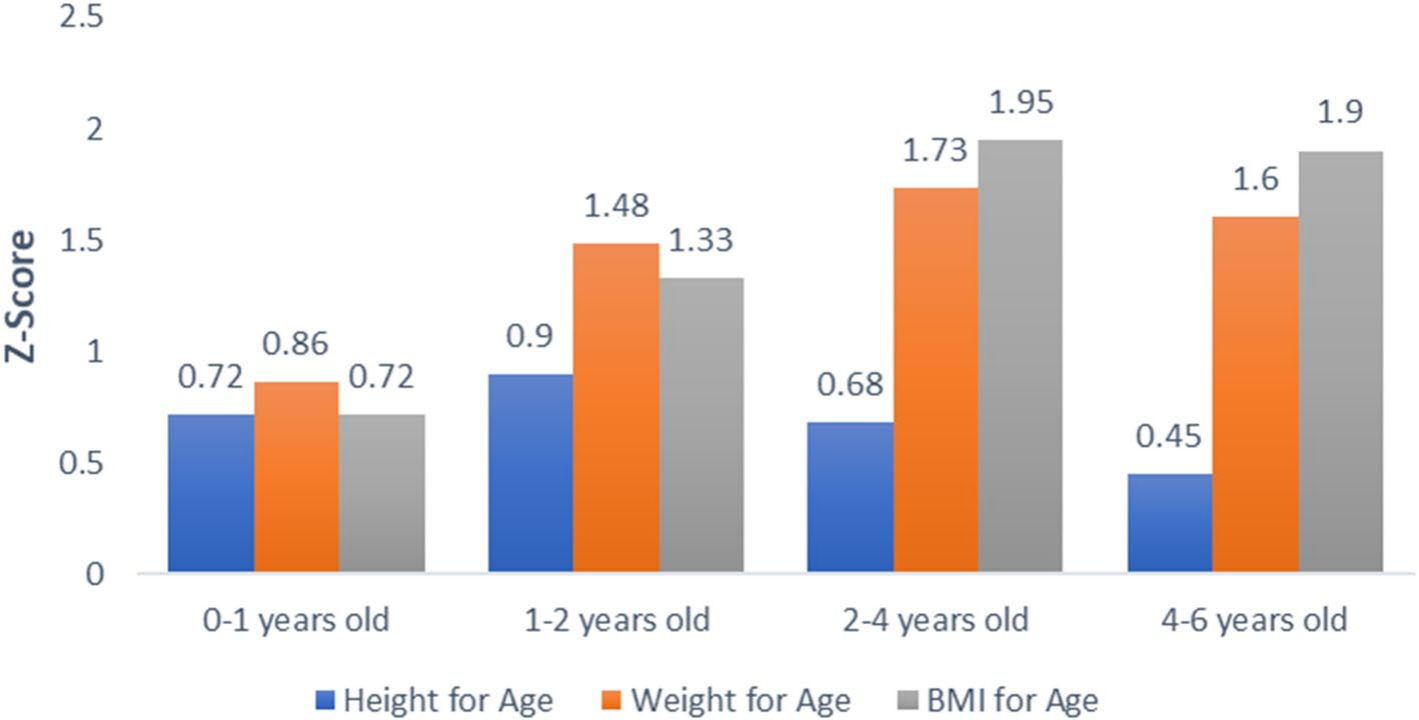
Mean Z-scores (height-for-age, weight-for-age, and BMI-for-age) at specific age ranges

Most mothers consumed the recommended supplements throughout pregnancy, and 66% of mothers reported smoking during pregnancy. Figure 1 shows average z-scores for four age categories. It is important to note that all of these average z-scores were above the WHO mean (0) and WAZ and BAZ indicated a positive trend with increasing age category. Only 1–1.5% of measurements were classified within the underweight and wasted category and only 3% were stunted, and no child consistently had measurements within these categories over repeated follow-up visits.

We constructed logistic regression models with GDM, high birthweight, and RWG as the outcome variables (Tables 2, 3, and 4). GDM was positively associated with maternal age (OR: 1.19; CI: 1.07–1.35) (Table 2). We considered RWG in 3 distinct models as an increase in z-score > 0.67, 1.28, and 2 SD, respectively, between two measurements from birth to six years of age. The logistic regression results of these models are shown in Table 4. There was an almost ten fold higher odds of RWG (> 2SD) if the child was exposed to GDM in utero (OR: 9.93; CI: 2.18–53.50) and a lower odds of RWG with higher birthweight per 1 lb (OR: 0.52; CI: 0.29–0.86) (Table 4).

**Table 2:** ^a^Logistic regression of factors associated with GDM

**Dependent: Gestational** **Diabetes (GDM)**		**No GDM**	**GDM**	**OR (univariable)**	**OR (multivariable)**
**Maternal Age**	Mean (SD)	24.9 (5.6)	31.0 (7.0)	1.17 (1.10–1.27)	1.19 (1.07–1.35)
**Pregnancy Weight Gain**	Less than 10% gain	52 (71.2)	21 (28.8)	-	-
	More than 10% gain	56 (81.2)	13 (18.8)	0.57 (0.26–1.25)	0.81 (0.19–3.37)
**Sex**	Boys	52 (74.3)	18 (25.7)	-	-
	Girls	67 (79.8)	17 (20.2)	0.73 (0.34–1.56)	2.11 (0.49–10.57)
**Cigarette Smoking during Pregnancy**	Non-smoker	44 (81.5)	10 (18.5)	-	-
	Smoker	69 (73.4)	25 (26.6)	1.59 (0.71–3.78)	0.92 (0.21–4.44)
**Parity**	Only Child	14 (73.7)	5 (26.3)	-	-
	Other Children	36 (75.0)	12 (25.0)	0.93 (0.29–3.37)	0.69 (0.10–4.57)

^a^Variables included in the multivariate models are those listed in the table, including maternal age, pregnancy weight gain (pre-pregnancy weight compared to 1 year post-pregnancy), child’s sex, cigarette smoking during pregnancy, and parity

**Table 3:** ^a^Logistic regression with odds ratios for high birthweight (> 4000 g)

**Dependent: Birthweight**		**Birthweight < 4000 g and > 2500 g**	**High Birthweight (> 4000 g)**	**OR (univariable)**	**OR (multivariable)**
**Maternal Age**	Mean (SD)	26.0 (6.3)	25.9 (6.8)	1.00 (0.93–1.07)	0.99 (0.91–1.07)
**Sex**	Boys	58 (84.1)	11 (15.9)	-	-
	Girls	62 (84.1)	16 (20.5)	1.36 (0.59–3.25)	1.43 (0.51–4.20)
**GDM**	No GDM	95 (84.1)	18 (15.9)	-	-
	GDM	23 (71.9)	9 (28.1)	2.07 (0.80–5.12)	2.89 (0.84–9.94)
**Cigarettes during pregnancy**	Non-smoker	37 (74.0)	13 (26.0)	-	-
	Smoker	80 (87.0)	12 (13.0)	0.43 (0.18–1.03)	0.49 (0.17–1.40)
**Pregnancy weight change**	Less than 10% change	60 (83.3)	12 (16.7)	-	-
	More than 10% weight gain	52 (80.0)	13 (20.0)	1.25 (0.52–3.01)	1.63 (0.57–4.93)

^a^Variables included in the multivariate models are those listed in the table, including maternal age, sex, GDM, cigarettes during pregnancy, and pregnancy weight change

**Table 4:** ^a^Multivariate logistic regression for rapid weight gain (> 0.67 SD gain, > 1.28 SD gain, and > 2 SD gain) from birth to 6 years

**Dependent: Rapid Weight Gain**		**No RWG**	**RWG > 0.67 SD**	**OR (univariable)**	**OR (multivariable)**
**Maternal Age**	Mean (SD)	26.7 (6.5)	24.9 (6.8)	0.96 (0.90–1.02)	0.92 (0.85–1.00)
**Child’s Sex**	Boys	18 (38.3)	29 (61.7)	-	-
	Girls	26 (48.1)	28 (51.9)	0.67 (0.30–1.47)	0.55 (0.21–1.35)
**GDM**	No GDM	37 (46.8)	42 (53.2)	-	-
	GDM	6 (30.0)	14 (70.0)	2.06 (0.74–6.31)	7.47 (1.68–46.22)
**Birthweight**	Mean (SD)	7.9 (1.1)	7.5 (1.2)	0.75 (0.52–1.06)	0.69 (0.45–1.02)
		**No RWG**	**RWG > 1.28 SD**	**OR (univariable)**	**OR (multivariable)**
**Maternal Age**	Mean (SD)	26.2 (6.2)	24.8 (7.5)	0.97 (0.91–1.03)	0.93 (0.85–1.00)
**Child’s Sex**	Boys	24 (51.1)	23 (48.9)	-	-
	Girls	39 (72.2)	15 (27.8)	0.40 (0.17–0.91)	0.35 (0.13–0.87)
**GDM**	No GDM	53 (67.1)	26 (32.9)	-	-
	GDM	9 (45.0)	11 (55.0)	2.49 (0.92–6.92)	6.02 (1.57–27.66)
**Birthweight**	Mean (SD)	7.8 (1.1)	7.6 (1.3)	0.87 (0.61–1.23)	0.85 (0.57–1.26)
		**No RWG**	**RWG > 2 SD**	**OR (univariable)**	**OR (multivariable)**
**Maternal Age**	Mean (SD)	25.5 (6.4)	26.3 (8.0)	1.02 (0.94–1.10)	0.95 (0.85–1.04)
**Child’s Sex**	Boys	35 (74.5)	12 (25.5)	-	-
	Girls	46 (85.2)	8 (14.8)	0.51 (0.18–1.36)	0.37 (0.10–1.24)
**GDM**	No GDM	68 (86.1)	11 (13.9)	-	-
	GDM	11 (55.0)	9 (45.0)	5.06 (1.70–15.28)	9.93 (2.18–53.50)
**Birthweight**	Mean (SD)	7.8 (1.1)	7.1 (1.3)	0.59 (0.36–0.93)	0.52 (0.29–0.86)

^a^Variables included in the multivariate analyses were maternal age, sex, gestational diabetes, and birthweight with rapid weight gain (RWG), defined in three different ways (> 0.67 SD, > 1.28 SD, > 2 SD) in the three models, as the dichotomous outcome

### Generalized additive models

**Fig. 2:**
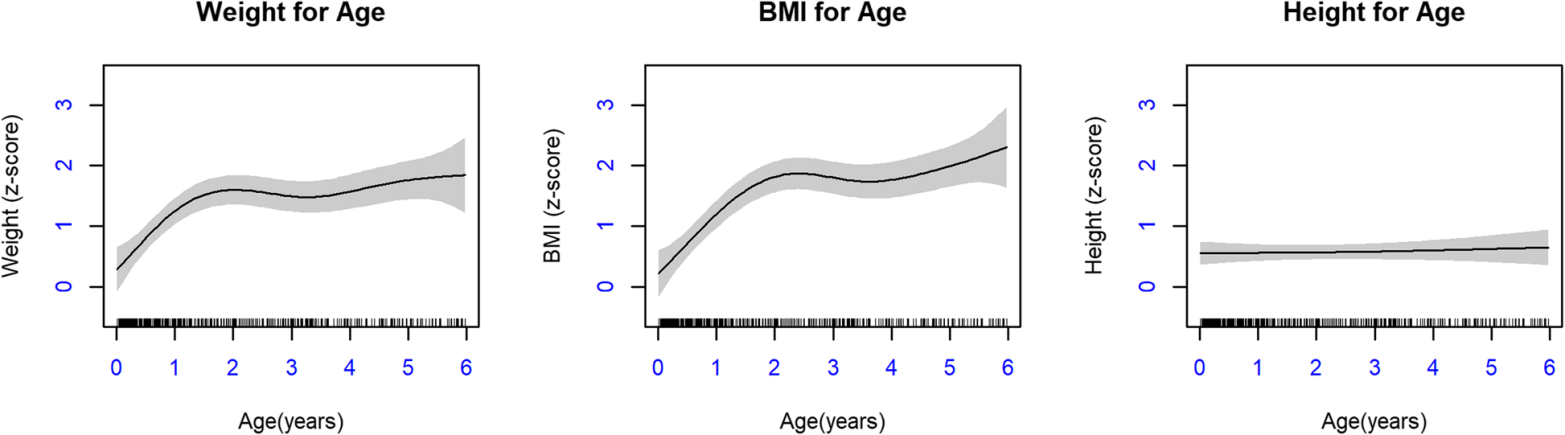
Overall Generalized Additive Models (GAMs) for Weight-for-age, BMI-for-age, and Height-for-age Z-scores

**Fig. 3:**
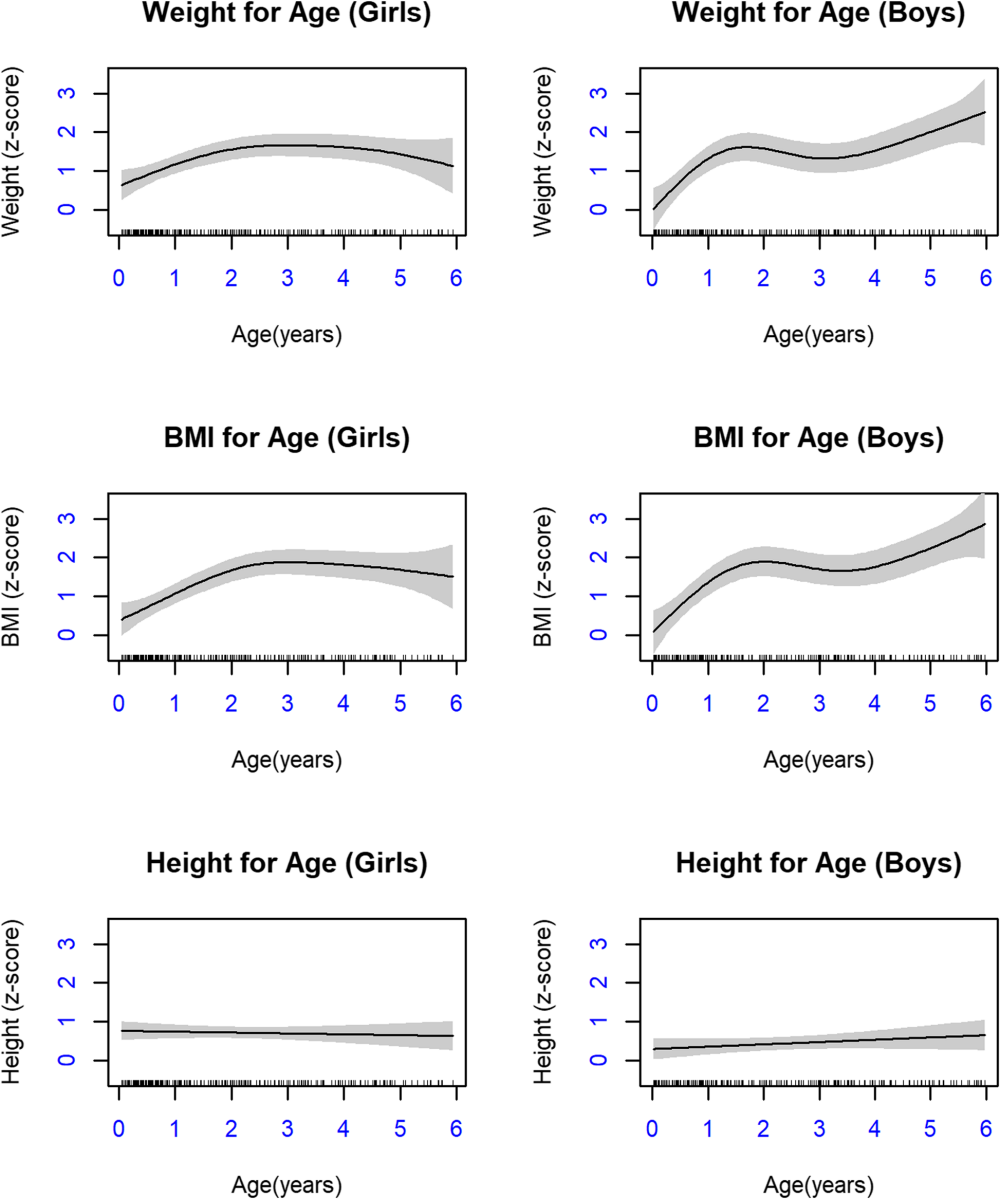
Generalized Additive Models (GAMs) for Weight-for-age, BMI-for-age, and Height-for-age z-scores for both boys and girls ages 0 to 6 years in Sandy Lake

We completed generalized additive models using WAZ, HAZ, and BAZ as the dependent variables and age as the time/independent variable. These overall models are shown in Fig. 2. Z-score trajectories were above the WHO mean (0) from birth to six years with an increase in weight-for-age and BMI-for-age z-scores with increasing age. Height-for-age z-score trajectories mirror the WHO population; however, Sandy Lake infants and children were approximately 0.5 SD taller. In addition to considering the overall growth trajectories for this population, we also completed separate models for boys and girls (Fig. 3).

Differences were observed between GAMs for boys and girls. For WAZ and BAZ trajectories, boys grew more quickly and had a greater increase in z-scores with increasing age up to age six. Girls’ WAZ and BAZ also increased with time, but plateaued around age three, although girls also had fewer data available at older ages, which may have contributed to imprecise results at these ages. Models for HAZ were linear, with the effective degrees of freedom in both models being close to one. This indicates that the pattern of HAZ growth was very similar to the WHO reference population, albeit approximately 0.5 SD higher for both boys and girls.

### Longitudinal associations of z-scores with health and social factors

**Fig. 4:**
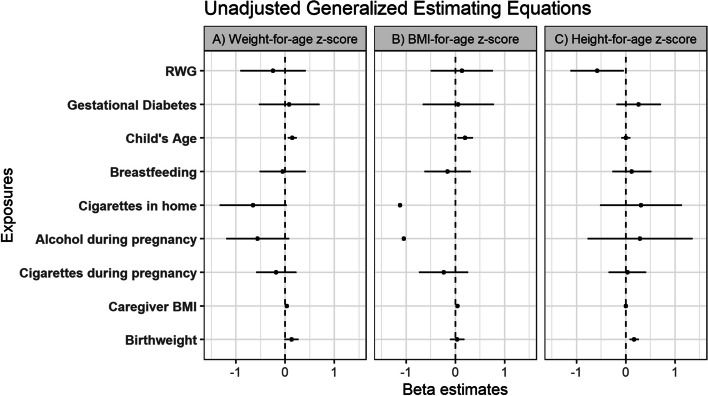
Generalized estimating equation estimates for early life health factors (% maternal weight change, bottle feeding, cigarettes in the home, other drugs during pregnancy, alcohol during pregnancy, cigarettes during pregnancy, birthweight, breastfeeding initiation, gestational diabetes, child’s age, and caregiver BMI) with **a**) WAZ as the outcome in separate unadjusted models **b**) BAZ as the outcome in separate unadjusted models and **c**) HAZ as the outcome in separate unadjusted models

**Fig. 5:**
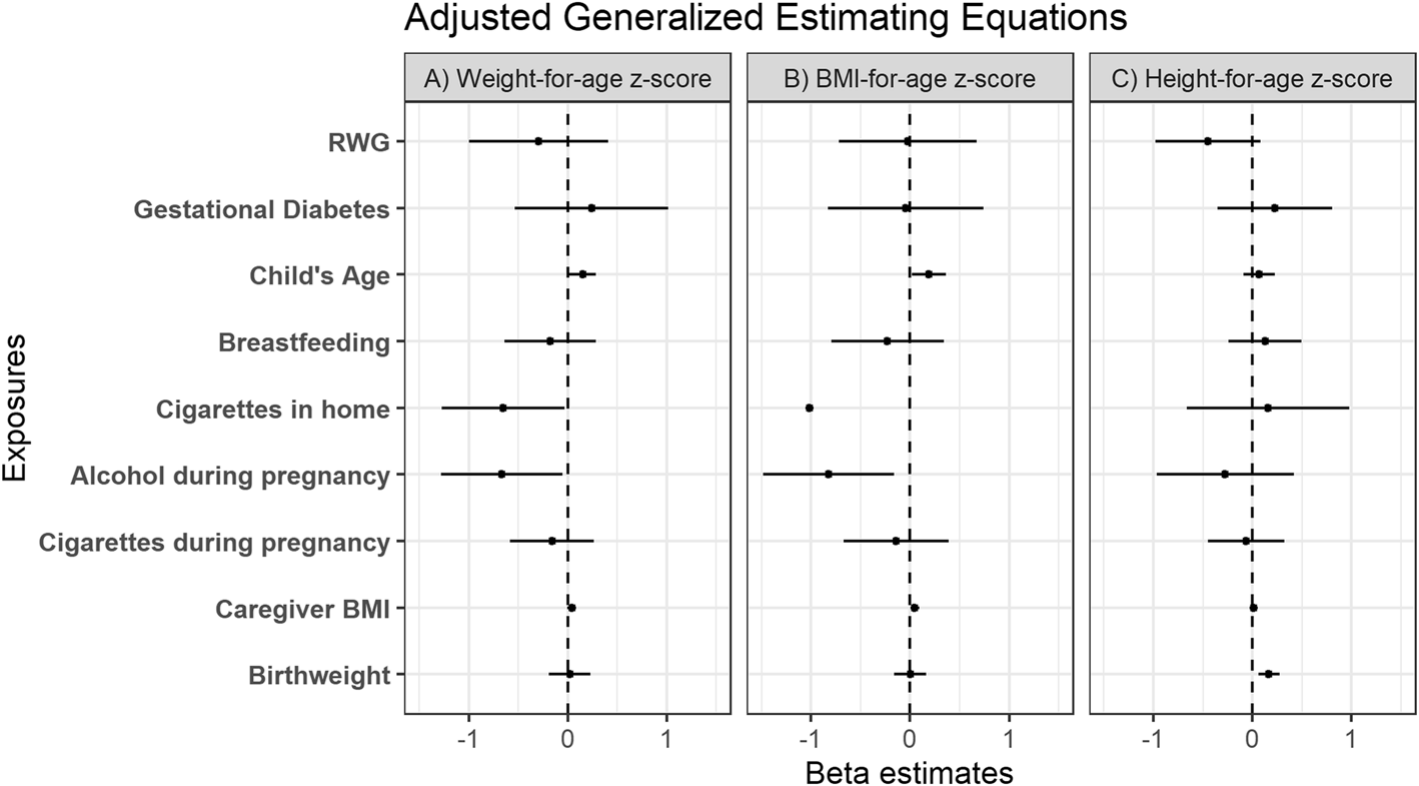
Generalized estimating equation estimates for early life health factors (% maternal weight change, bottle feeding, cigarettes in the home, other drugs during pregnancy, alcohol during pregnancy, cigarettes during pregnancy, birthweight, breastfeeding initiation, gestational diabetes, child’s age, and caregiver BMI) adjusted for maternal age, sex, employment status, child’s age, birthweight, diabetes in pregnancy, and breastfeeding status with **a**) WAZ as the outcome **b**) BAZ as the outcome and **c**) HAZ as the outcome

Generalized estimating equations were used to evaluate longitudinal associations between markers of growth (eg. WAZ, BAZ and HAZ) and factors determined through health questionnaires (Figs. 4, 5). WAZ was positively associated with child’s age in both the unadjusted (β: 0.145; CI: 0.056–0.23) and adjusted (β: 0.15; CI: 0.013–0.29) models, which was consistent with the GAM results which illustrated an increasing WAZ with increasing age (Fig. 3). In the adjusted model, WAZ was also negatively associated with alcohol during pregnancy (β: -0.67; CI: -1.28-(-0.06)) and having someone who smoked cigarettes in the home (β: -0.65; CI: -1.28-(-0.03). Similar to WAZ, BAZ was positively associated with child’s age in both the unadjusted (β: 0.20; CI: 0.04–0.36) and adjusted (β: 0.19; CI: 0.02–0.36) models, also consistent with the GAM analysis shown earlier in Fig. 3. BAZ was also positively associated with caregiver BMI (β: 0.049; CI: 0.004–0.093), and negatively associated with alcohol during pregnancy (β: -0.82; CI: -1.48-(-0.16)) in the adjusted model. HAZ was positively associated with birthweight in both the adjusted (β: 0.17; CI: 0.06–0.27) and unadjusted (β: 0.17; CI: 0.08–0.27) models and negatively with RWG in the unadjusted model (β: -0.58; CI: -1.21-(-0.04)).

## Discussion

This growth project is part of a larger research initiative on diabetes prevention within Sandy Lake, Ontario, and aligns with clinical and public health concerns and community priorities related to childhood risk factors. Sandy Lake infants and children who participated in this study were taller and heavier than infants and children within the WHO reference population. Participants’ WAZ and BAZ increased with increasing age, indicating an increasing gap between the WHO mean and Sandy Lake children over time. Canadian population data from the Canadian Community Health Survey (2004–05) and the Canadian Health Measures Survey (2009–2013) also showed higher BAZ compared to the WHO reference population, although the mean z-score for this Sandy Lake cohort was still more than 1 SD above the Canadian population data [42]. Data from the 2002/03 First Nations Regional Health Survey in Canada also noted a high prevalence of overweight and obesity in early years, with high prevalence also noted throughout the life span [43]. Although Sandy Lake infants generally start with higher birthweights compared with non-Indigenous groups, greater weight differences are seen as children grow over time, indicating that other early life factors may be contributing to faster growth. The prevalence of GDM in Sandy Lake (21.1%) falls within prevalence recorded by other Canadian studies showing rates among First Nations communities ranging from 8.5% to 27% [44]; however, these rates are higher than the general Ontario population (5.6%) [45]. Similarly, prevalence rates for hypertension, anemia, and smoking during pregnancy are consistent with other data among First Nations women living in Canada [46]. Despite the similar prevalence findings for cigarette smoking during pregnancy, these rates are markedly above the prevalence reported for non-Indigenous groups in Canada and pose a health risk to infants and mothers [46]. Studies highlight that cigarette smoking during pregnancy can result in low birthweight; however, data is mixed on subsequent child adiposity [5]. Our study did not find an association between smoking during pregnancy and birthweight; however, cigarette smoking in the home was negatively associated with WAZ and BAZ.

While this study emphasizes the impact of overnutrition on this population, undernutrition is a primary concern impacting infant and child growth within other Indigenous populations globally [47]; however, this study identified only 1–1.5% of measurements as classified within the underweight and wasted category and only 3% as stunted, and no children had measurements consistently within these categories over repeated follow-up visits. This observation highlights that children’s energy and other macronutrient needs are being met despite documented challenges within the community, including high food prices, a changing climate that can limit access to traditional foods, unemployment, health provider turnover, and distance from services [24, 25]. For many Indigenous communities, colonization has resulted in a high burden of chronic disease, limited access to quality health services, food insecurity and a lack of food sovereignty, unemployment, poverty, and culturally unsafe experiences [11, 24, 43, 48, 49]. This is reflected in the high unemployment rate within this cohort, as well as a high prevalence of maternal health concerns, like GDM. Although we had limited data to precisely document the impact of these factors, it will be important for future research projects to evaluate in more detail the impact of these phenomena.

Previous studies have noted sex differences in growth patterns in infancy and early childhood [50]; however, these differences vary by population. Tumilowicz et al. [51] followed growth among Indigenous infants and young children living in Guatemala and identified that girls were bigger and that mothers perceived boys as requiring more nutrition to meet needs. In contrast, a US cohort followed non-Indigenous children from 1 to 10 years of age and identified that boys had higher BMI for age at 15 months, but had more extreme decline and rebound growth thereafter up to ten years [18]. In the current study, we observed that boys had more rapid growth from birth to two years of age, followed by another increase in WAZ and BAZ from four to six years; whereas although girls also had an increase in WAZ and BAZ in the early years, this incline slowly continued until age three, followed by no further growth velocity differences compared to the WHO population. Our data suggest that boys may have higher WAZ and BAZ leading into childhood; however, further work is needed that considers growth throughout childhood and into adolescence. In consideration of the significant burden of diabetes on First Nations women, we might expect that girls would have early signs of higher risk (higher WAZ and BAZ); however, this is not what we observed and highlights a need to better understand the role of paternal health intergenerationally. HAZ was very similar between boys and girls with similar trajectory to the WHO population, although girls were longer in the first two years and appear to enter childhood as taller; however, we observed a wider confidence interval at this time related to fewer data points from 5–6 years for girls, which resulted in an imprecise estimate for the trajectory for this interval.

## Limitations

The Canadian Paediatric Society recommends that Indigenous groups follow the same growth monitoring as is recommended for all Canadian children and BMI is supported as a growth measure given the risk for overweight and obesity [52]. However, scholars have raised concerns about the use of BMI as a reliable growth measure [53], particularly for Indigenous groups, as there is a lack of population-relevant reference values and Indigenous children were not included in the WHO growth standards. In this study, we used WHO z-scores, which are based on data of healthy infants and children from six countries. Willows et al. [21] noted differences between CDC and IOTF references for Cree children, questioning the use of these measures for this population; therefore, we chose to use the WHO references, but it is not yet clear whether these references are appropriate for Indigenous groups. Further work is needed to consider raw growth data for Indigenous groups recognizing that these data have not been included in current growth standards and comparison between growth references would help to clarify which and if these references are appropriate.

It is also important to highlight that despite significant effort for community recruitment in this study, our sample size is limited. Although we had 194 children enrolled in this study, values were missing for a variety of variables and repeat growth measurements. This influenced the type of analyses that were feasible for this study and limited sub-analysis. In addition, this study is community-specific, and results may differ for other Indigenous groups. Further community-led research that considers early life factors and growth trajectories is needed within Indigenous groups in Canada to better understand and address intergenerational health.

## Conclusion

This study fills an important gap in the literature, adding to the one study published to date on growth trajectories in an Indigenous community in Canada [21], and providing an example of Indigenous growth trajectories; the current results build on this work with further discussion of factors influencing growth. Despite a challenging socio-ecological environment, caregivers in Sandy Lake are working hard to ensure healthy growth of infants and children. There is a need for more community support, including interventions and policies that address root causes of overweight and diabetes. Ongoing work by the SLHDP is focused on community- driven initiatives that involve children and youth and consider maternal and infant health and nutrition [27]. Indigenous methodologies, including a focus on intergenerational health and access to traditional food and medicine, must drive key research priorities in this area and ensure culturally safe research and healthcare.

## Data Availability

Additional summary data for this study is available upon request and with community approval. Individual participant data is not available for sharing unless approval is provided by Sandy Lake First Nation, abiding by community data sovereignty guidelines. Please contact Anthony.hanley@utoronto.ca for inquiries.

## Abbreviations

BAZ: Body Mass Index for Age Z-Score
BMI: Body Mass Index
CCHS: Canadian Community Health Survey
CHMS: Canadian Health Measures Survey
CDC: Centers for Disease Control and Prevention
DOHaD: Developmental Origins of Health and Disease
GAM: Generalized Additive Models
GDM: Gestational Diabetes Mellitus
HAZ: Height for Age Z-Score
IOTF: International Obesity Task Force
RHS: Regional Health Survey
RWG: Rapid Weight Gain
SD: Standard Deviation(s)
SLHDP: Sandy Lake Health and Diabetes Project
T2DM: Type 2 Diabetes Mellitus
WAZ: Weight for Age Z-Score
WHO: World Health Organization

## References

[1] Barker DJP, Bagby SP (2005). Developmental antecedents of cardiovascular disease: a historical perspective. J Am Soc Nephrol..

[2] Rose G (1964). Familial patterns in ischaemic heart disease. Br J Prev Soc Med..

[3] Barker DJP, Osmond C (1986). Infant mortality, childhood nutrition, and ischaemic heart disease in england and wales. The Lancet..

[4] Agarwal P, Morriseau TS, Kereliuk SM, Doucette CA, Wicklow BA, Dolinsky VW (2018). Maternal obesity, diabetes during pregnancy and epigenetic mechanisms that influence the developmental origins of cardiometabolic disease in the offspring. Crit Rev Clin Lab Sci..

[5] McNamara BJ, Gubhaju L, Chamberlain C, Stanley F, Eades SJ (2012). Early life influences on cardio-metabolic disease risk in aboriginal populations–what is the evidence? A systematic review of longitudinal and case-control studies. Int J Epidemiol.

[6] Indigenous Corporate Training Inc. What is the Seventh Generation Principle?. 2020 [cited 2022 May 16]. Available from: https://www.ictinc.ca/blog/seventh-generation-principle.

[7] Reading CL, Wien F. Health Inequalities and Social Determinants of Aboriginal Peoples’ Health. National Collaborating Centre for Aboriginal Health; 2009.

[8] Crowshoe L, Dannenbaum D, Green M, Henderson R, Hayward MN, Toth E (2018). Type 2 Diabetes and Indigenous Peoples. Can J Diabetes.

[9] Dyck R, Osgood N, Lin TH, Gao A, Stang MR (2010). Epidemiology of diabetes mellitus among First Nations and non-First Nations adults. Can Med Assoc J.

[10] Park J (2021). Mortality among First Nations people, 2006 to 2016. Health Rep.

[11] Jacklin KM, Henderson RI, Green ME, Walker LM, Calam B, Crowshoe LJ (2017). Health care experiences of Indigenous people living with type 2 diabetes in Canada. Can Med Assoc J.

[12] Blotsky AL, Rahme E, Dahhou M, Nakhla M, Dasgupta K (2019). Gestational diabetes associated with incident diabetes in childhood and youth: a retrospective cohort study. CMAJ.

[13] Wicklow BA, Sellers EAC, Sharma AK, Kroeker K, Nickel NC, Philips-Beck W (2018). Association of Gestational Diabetes and Type 2 Diabetes Exposure In Utero With the Development of Type 2 Diabetes in First Nations and Non-First Nations Offspring. JAMA Pediatr..

[14] Dyck RF, Karunanayake C, Pahwa P, Osgood ND. The hefty fetal phenotype hypothesis revisited: high birth weight, type 2 diabetes and gestational diabetes in a Saskatchewan cohort of First Nations and non-First Nations women. J Dev Orig Health Dis. 2019;10(1):48–54. 10.1017/S2040174417000988. 10.1017/S2040174417000988 29271332

[15] Geserick M, Vogel M, Gausche R, Lipek T, Spielau U, Keller E (2018). Acceleration of BMI in Early Childhood and Risk of Sustained Obesity. N Engl J Med.

[16] Rotevatn TA, Overgaard C, Melendez-Torres GJ, Mortensen RN, Ullits LR, Høstgaard AMB (2019). Infancy weight gain, parental socioeconomic position, and childhood overweight and obesity: a Danish register-based cohort study. BMC Public Health.

[17] Pizzi C, Cole TJ, Richiardi L, dos-Santos-Silva I, Corvalan C, De Stavola B (2014). Prenatal Influences on Size, Velocity and Tempo of Infant Growth: Findings from Three Contemporary Cohorts. PLoS One..

[18] Boyer BP, Nelson JA, Holub SC (2015). Childhood body mass index trajectories predicting cardiovascular risk in adolescence. J Adolesc Health Off Publ Soc Adolesc Med.

[19] Eny KM, Maguire JL, Dai DWH, Lebovic G, Adeli K, Hamilton JK, et al. Association of accelerated body mass index gain with repeated measures of blood pressure in early childhood. Int J Obes. 2019;43(7):1354–62. 10.1038/s41366-019-0345-9. 10.1038/s41366-019-0345-9 PMC6760600 30940913

[20] Eny KM, Chen S, Anderson LN, Chen Y, Lebovic G, Pullenayegum E (2018). Breastfeeding duration, maternal body mass index, and birth weight are associated with differences in body mass index growth trajectories in early childhood. Am J Clin Nutr.

[21] Willows ND, Johnson MS, Ball GDC (2007). Prevalence Estimates of Overweight and Obesity in Cree Preschool Children in Northern Quebec According to International and US Reference Criteria. Am J Public Health.

[22] Walker JD, Slater M, Jones CR, Shah BR, Frymire E, Khan S (2020). Diabetes prevalence, incidence and mortality in First Nations and other people in Ontario, 1995–2014: a population-based study using linked administrative data. CMAJ.

[23] McQueen K, Sieswerda LE, Montelpare W, Dennis C (2015). Prevalence and Factors Affecting Breastfeeding Among Aboriginal Women in Northwestern Ontario. J Obstet Gynecol Neonatal Nurs.

[24] First Nations Information Governance Centre. National Report of the First Nations Regional Health Survey Phase 3: Volume Two. First Nations Information Governance Centre; 2018.

[25] Government of Canada SC. Aboriginal Community Data Initiative Portrait, 2016 Census – Sandy Lake First Nation. 2020. Available from: https://www12.statcan.gc.ca/census-recensement/2016/dp-pd/abpopprof/infogrph/infgrph.cfm?LANG=E&DGUID=2016C1005246&PR=35.

[26] Hanley A, Harris SB, Barnie A, Gittlesohn J, Wolever TMS, Logan A (1995). The Sandy Lake Health and Diabetes Project: Design, methods and lessons learned. Chronic Can.

[27] Kakekagumick KE, Naqshbandi Hayward M, Harris SB, Saksvig B, Gittelsohn J, Manokeesic G, et al. Sandy Lake Health and Diabetes Project: A Community-Based Intervention Targeting Type 2 Diabetes and Its Risk Factors in a First Nations Community. Front Endocrinol. 2013;4:170. 10.3389/fendo.2013.00170. 10.3389/fendo.2013.00170 PMC3824247 24302919

[28] Jasper EA, Cho H, Breheny PJ, Bao W, Dagle JM, Ryckman KK (2021). Perinatal determinants of growth trajectories in children born preterm. PLoS One.

[29] Office of Professional and Educational Development, Memorial University of Newfoundland. Rourke Baby Record. The Rourke Baby Record. Available from: https://www.rourkebabyrecord.ca/rbr2020/default.

[30] Carsley S, Borkhoff CM, Maguire JL, Birken CS, Khovratovich M, McCrindle B (2015). Cohort Profile: The Applied Research Group for Kids (TARGet Kids!). Int J Epidemiol.

[31] Posit Team. RStudio: Integrated Development Environment for R [Internet]. Boston MA: Posit Software, PBC; 2023. Available from: http://www.posit.co/.

[32] WHO Multicentre Growth Reference Study Group (2006). WHO Child Growth Standards based on length/height, weight and age. Acta Paediatr Oslo Nor 1992 Suppl.

[33] World Health Organization. WHO Child Growth Standards: Length/height-for-age, weight-for-age, weight-for-length, weight-for-height and body mass index-for-age: Methods and Development. World Health Organization; 2006. Available from: https://www.who.int/publications/i/item/924154693X.

[34] Zhang X, Tilling K, Martin RM, Oken E, Naimi AI, Aris IM (2019). Analysis of ‘sensitive’ periods of fetal and child growth. Int J Epidemiol.

[35] World Health Organization (1995). Physical status : the use of and interpretation of anthropometry, report of a WHO expert committee.

[36] A health professional’s guide for using the new WHO growth charts. Paediatr Child Health [Internet]. 2010 [cited 2022 Jan 14];15(2):84–90. Available from: https://www.ncbi.nlm.nih.gov/pmc/articles/PMC2865941/. 10.1093/pch/15.2.84 PMC2865941 21286296

[37] Hastie T, Tibshirani R (1990). Generalized Additive Models.

[38] First Nations Information Governance Centre (FNIGC). First Nations Regional Health Survey (RHS) 2008/10: National Report on Adults, Youth and Children living in First Nations Communities [Internet]. Ottawa: FNIGC; 2012. Available from: https://fnigc.ca/wp-content/uploads/2020/09/ccd66b67e9debb2c92f4a54703e1d050_First-Nations-Regional-Health-Survey-RHS-2008-10-National-Report.pdf.

[39] Monteiro POA, Victora CG (2005). Rapid growth in infancy and childhood and obesity in later life–a systematic review. Obes Rev Off J Int Assoc Study Obes.

[40] Wang G, Johnson S, Gong Y, Polk S, Divall S, Radovick S (2016). Weight Gain in Infancy and Overweight or Obesity in Childhood across the Gestational Spectrum: a Prospective Birth Cohort Study. Sci Rep.

[41] Twisk JWR. Applied Longitudinal Data Analysis for Epidemiology: A Practical Guide (Second Edition). Cambridge: Cambridge University Press; 2013. p. 57–68. 10.1017/CBO9781139342834.

[42] Rodd C, Sharma AK (2016). Recent trends in the prevalence of overweight and obesity among Canadian children. CMAJ..

[43] First Nations Centre (2005). First Nations Regional Longitudinal Health Survey (RHS) 2002/03.

[44] First Nations Centre des Premières Nations. Gestational Diabetes and First Nations Women: A literature Review. First Nations Centre, National Aboriginal Health Organization; 2009. Available from: https://fnim.sehc.com/getmedia/a7198c67-da87-44e9-9cc4-1d681303fef8/Gestational_Diabetes_LitReview_2009.pdf.aspx?ext=.pdf.

[45] Feig DS, Hwee J, Shah BR, Booth GL, Bierman AS, Lipscombe LL (2014). Trends in incidence of diabetes in pregnancy and serious perinatal outcomes: a large, population-based study in Ontario, Canada, 1996–2010. Diabetes Care.

[46] Gould GS, Patten C, Glover M, Kira A, Jayasinghe H (2017). Smoking in Pregnancy Among Indigenous Women in High-Income Countries: A Narrative Review. Nicotine Tob Res.

[47] Ferreira AA, Welch JR, Cunha GM, Coimbra CEA (2016). Physical growth curves of indigenous Xavante children in Central Brazil: results from a longitudinal study (2009–2012). Ann Hum Biol.

[48] Cidro J, Martens TR, Zahayko L, Lawrence HP (2018). First foods as Indigenous food sovereignty: Country foods and breastfeeding practices in a Manitoban First Nations community. Can Food Stud Rev Can Études Sur Aliment.

[49] Bhawra J, Cooke MJ, Hanning R, Wilk P, Gonneville SLH (2015). Community perspectives on food insecurity and obesity: Focus groups with caregivers of metis and Off-reserve first nations children. Int J Equity Health.

[50] Shah B, Tombeau Cost K, Fuller A, Birken CS, Anderson LN (2020). Sex and gender differences in childhood obesity: contributing to the research agenda. BMJ Nutr Prev Health.

[51] Tumilowicz A, Habicht JP, Pelto G, Pelletier DL (2015). Gender perceptions predict sex differences in growth patterns of indigenous Guatemalan infants and young children. Am J Clin Nutr.

[52] Growth assessment in Aboriginal children (2004). Is there need for change?. Paediatr Child Health.

[53] Bogin B (2020). Patterns of Human Growth.

